# Misalignments of values and preferences: Finding an ideal elder care arrangement

**DOI:** 10.1136/jme-2024-110559

**Published:** 2025-05-07

**Authors:** Yi Jiao Angelina Tian, Tenzin Wangmo, Julian Savulescu

**Affiliations:** 1 Institute for Biomedical Ethics, University of Basel, Basel, Switzerland; 2 Centre for Biomedical Ethics, National University of Singapore, Singapore; 3 Uehiro Oxford Institute, University of Oxford, Oxford, UK

**Keywords:** Aged, Ethics, Health Personnel, Information Technology

## Abstract

The ageing of the global population prompts many countries to appropriately allocate healthcare resources that ensure adequate elder care. Nevertheless, the shortages in and burdens of professional care continue to persist. Assistive and remote monitoring technologies for home-use support professional carers in providing care to older persons. With secondary analyses of semistructured interviews with 27 older persons and 23 professional carers in Switzerland, we examined their reasons and expectations for accepting or rejecting technologies in elder care contextualised by their moral outlooks on care, life, death and the deterioration from age. Whereas some appreciate the opportunities for greater safety and reassurance from technologies, others may see the alerts as burdensome and the interventions superfluous. We argue that dissatisfaction in professional care may result from a misalignment of the subjective values between the carer and older person. This may exacerbate the problem of appropriate care provision and disrupt the potential of technologies to benefit older persons. An ideal caregiving arrangement may be found when their values do align. We argue that there exists intrinsic value to finding an alignment using the capabilities approach, followed by reflections on autonomy and privacy. Recommendations are offered to practically enable this alignment, with limits set to ensure adequate access to care. With the increasing enthusiasm for technical solutions in professional elder care, this paper contributes a novel perspective by presenting two reasons for inefficiencies in reducing care burdens that are linked to the alignment of core moral outlooks and the realisation of capabilities.

## Introduction

 The global ageing population demands more caregiving resources from governments and families to ensure that older persons receive an appropriate quality of care to age with dignity and comfort. In Switzerland, people over the age of 65 years have already reached almost 20% of the population in 2020 and will climb to around 30% in 2060.^1^ Ageing involves a multifaceted array of physical, psychological, emotional, cognitive and social changes that may contribute to decreased independence and greater reliance on professional care often provided in the forms of home-based or community-based care.^2^ Nevertheless, this greater demand for professional care challenges an elder care industry that is already mired with staffing shortages. Professional carers report high care burdens, income dissatisfaction and insufficient contact with their own families.^3^ They commonly suffer from fatigue and burnout, self-neglect and lack of appreciation by their care recipients.^4^ During the COVID-19 pandemic, professional carers shouldered even more to compensate for the lack of familial contact.^5^

Smart homes have gradually gained attention as a solution in elder care. Through a collection of devices worn on the body and installed on appliances or flooring in the home, an interoperable network may be established to provide more information about the older resident. Alarms may be activated by the older person or automatically triggered when sensors detect falls, changes in vital signs or movements, as well as any environmental changes in the home. These functions have the potential to alleviate care burdens by promising a temporary reduction in the need for constant human supervision, while improving chances for older persons to receive care when necessary. A wealth of empirical studies has been conducted on their capabilities to meet the existing needs,^6 7^ the opportunities and barriers for acceptance^8 9^ and their ethical concerns.^10^,^12^

Nevertheless, research does not yet show their clear benefits in professional care.^13^ While some articles did cite a reduction in care burdens from integrating technologies,^14 15^ other studies, interestingly mostly from professional carers, relay concerns for increased workload.^16^,^21^ Older persons were worried about technologies burdening their family members and opted for transferring the work to professional carers, which ultimately circled back to professional care burdens.^22^ More research is necessary in the context of an ageing population to specifically understand why and how these technologies may more effectively improve current professional care challenges.

Our primary analysis of a set of qualitative interviews with older persons and carers in Switzerland revealed a wealth of benefits, barriers and ethical concerns of smart home technologies in elder care^823^,^25^ . This paper specifically addresses the issue of professional caregiving burdens, where we conducted a secondary analysis to explore some of these reasons behind the inefficiencies of technologies and offer an argument on how technologies could be better implemented in professional care connected to moral outlooks and values.

## Methods

### Ethics approval

The data in this paper arise from a larger project that was approved by the Ethics Commission of Northwest and Central Switzerland (EKNZ). Participants received an information document that detailed research purposes, the rights to withdrawal, data protection measures, etc. All recordings and transcripts were stored separately from documents with basic participant demographics. Access for all data and documents was limited to project-relevant personnel. Participants signed a consent form after all of their questions and concerns were addressed in the presence of an interviewer.

### Participant recruitment

We used a purposive snowballing strategy with several rounds of recruitment. Recruitment flyers were distributed in Spitex (hospital-external home care), meal delivery services, nursing homes and assistive living facilities. Participants were encouraged to share the project details with others that fitted our criteria as follows: (1) older persons who were 65 years or above without immediate needs for hospitalisation, (2) professional carers who were financially compensated for providing elder care and (3) informal or familial carers, who were uncompensated for their care work.

### Data collection

Two female Swiss-German interviewers trained in qualitative methodology completed interviews between September 2021 and November 2022. One has since completed her Ph.D. education in biomedical ethics and the other pursuing her medical degree. The location of the interviews took place at the participants’ choosing, usually in their homes or cafes. The interviewers abided by the rules of mask-wearing, social distancing and enabled virtual meetings whenever requested.

The interviews followed the same structure. Participants were first prompted to elaborate on their current care: Older persons discussed their care needs and how their caregiving experiences were, while professional carers were asked about their day-to-day care work, obligations and experiences. Thereafter, participants were shown pictures of various technologies including the assistive button, smart wearable, ambient and visual sensors. For each technology, the interviewer questioned and encouraged participants to speak about any first impressions or previous experiences of the technologies, their perceived benefits or barriers to adoption and ethical concerns.

The project includes a total of 60 interviews. Excluding 16 interviews with familial carers, 44 were analysed for this paper (see table 1). The mean duration for the included interviews was 98 min (range 51–187). Only one interview was virtual.

**Table 1:** Participant Demographics

	Older persons (n=27)	Professional carers (n=23)
Gender ratio (F:M)	15:12	19:4
Mean age (F:M)	90.2:84.0	45.6:43.3
Setting of care	Homes: 13	Home care: 9
	Nursing homes: 10	Nursing homes: 9
	Assisted living: 4	Other: 5

### Data analysis

Recordings were transcribed into High German by the two interviewers. The research team discussed the first few transcripts of each population group in a round-table manner to establish the foundations of a coding tree. Independent coding was conducted thereafter via the software MAXQDA. After refining the code system, the first author then exported^1^ the codes and quotations for this paper onto a Microsoft Excel document for paper-specific analysis. The data for this paper essentially comprised participants’ reception or provision of professional care, how smart and assistive monitoring technologies impacted professional care, as well as expressions of values relevant to care and ageing. General technology acceptance, an overview of ethical issues such as privacy, trust etc, as well as data from the familial unpaid carers is published in other articles from the same project^823^,^26^ ^25^. This paper uses reflexive thematic analysis,^27^ where the first author organised the data coherently into themes that addressed the aims of this study. The results were then shared with the coauthors, who provided further rounds of feedback for iterative rounds of reorganisation of themes and subthemes.

## Findings

### The care context

Professional carers pointed to care work that they would have liked to but could not provide, either due to a lack of time, administrative tasks or the pressing need to get to another client. The staffing shortage was an endless and increasingly becoming an unsolvable problem, as a home carer put it: “*The question we’re asking ourselves more and more every day: Who is going to do all this?*” Professional carers felt satisfaction at being able “*bake with the residents or go for a walk,*” which became increasingly more difficult as less time for social or interpersonal care was available.

Personnel shortages meant longer waiting times, as one nursing home participant expressed “*You have to be patient. We have to be happy that we have someone to do it*.” While there was a lack of continuity of care or interpersonal contact, older persons appreciated those who could have time to chat a bit more and were kinder or more considerate enough to try and “*put on a friendly face”* (another older person). Unfortunately, many professional carers in the nursing homes seem “*a bit uninterested and do what they have to do very quickly”* before leaving the room immediately.

### Misaligning preferences and values

Participants held varying preferences towards technologies and values or expectations on professional care, life, death and ageing. While some appreciated the functions of technologies to alert them to health abnormalities and promote safety, others perceived alerts to be burdensome and superfluous to the ageing process. This theme explores two misalignments of the preferences for technologies and moral values between the professional carer and the older person that are relevant to professional elder care burdens.

#### Misalignment 1: Providing more technology-supported care than preferred by older persons

One misalignment may occur as such: The professional carers are motivated to use technologies to improve health and safety, but the older persons may prefer to stay independent and respect a natural progression of ageing. One nursing home carer expressed worry when residents fall and cannot call loud enough for help: “*Does the person really have to wait three or four hours alone with the injury?”* in which cases the alerts may be both reassuring and life-saving (also see box 1, quote 1). Behind these expressions of acceptance, professional carers seemed to hold broader moral claims for the obligation to keep older persons safe and protected from further harm. For these carers, technologies could improve care burdens as it allowed them to step away momentarily without worrying that older persons are at danger of harm (also see box 1, quote 3).

Box 1 **Additional quotes**

1. PCN7: Mostly when you're at home or alone in the apartment, nobody hears you. Or it's not so loud, or I have another resident who also said: “I had to shout so much, I didn't have any energy left at the end.” And then sometimes it’s a matter of luck that they are found.
2. OPH5: I'm actually ready. I talk to my husband a lot, I have a photo back there, a big one, and then I talk to him.
3. PCN4: It's a huge relief. I remember working in nursing at a time when it wasn't used to this extent, because [technologies] weren’t used like that. […] I used to experience my team colleagues in the night watch or in services where there are no longer so many employees, like "Oh, is he climbing over the bed rail, yes, no?", "Is something happening there?", you always had the worry, you did the rounds several times, looked, you are under greater tension.
4. PCN8: How far should we go? We also have many residents who actually want to die, and who perhaps don't want to—and who also write in their living will: “No resuscitation and no hospital transfer”, and who then also say, "You know, maybe I'll fall over and then I won't be able to get up.”

Nevertheless, older persons did not find these alerting capabilities to be always ideal. The generation of data and the focus on a promotion of health was perceived as unnecessary at this stage. If they felt subjectively in good health, there would not be a need to continuously monitor and alert carers for assistance. As one older person living at home expressed that ageing is a natural process of deterioration and dying is no longer something to be feared: “*And now I'm going to slow down anyway* […and] *there’s nothing you can do when the hour strikes* (also see quote 2). One older person in her 90s living alone at home expressed that she was content with getting through to her age and accepted the possibility of sudden falls: “*And if I fall out… I just fall out.”* When asked about whether they would autonomously decide to incorporate technologies that allowed alerts to carers, older persons preferred to delay them and remain independent partly because of a reluctance to become a burden to others. One nursing home resident admitted that she had a “*bad reputation* [for] *never ringing the bell”* as she did not like *ringing for nothing if* [she] *dropped a handkerchief or something,* [she doesn’t] *have to have someone to jump in and pick it up*. One should first take self-responsibility, rather than always expecting help.

When technologies are used in a pair of professional carers and older persons with such contrasting views, a misalignment occurs that may not improve caregiving burdens. A nursing home carer described a bedside pressure mat that alerts the carers when older persons get off from the bed to prevent wandering behaviours or night-time restlessness. Nevertheless, these mats become meaningless “*if the resident doesn't want it, then they find a way around it, and it’s usually very acrobatic things that they do”* to avoid triggering the emergency function by going around the mats and getting off from the foot of the bed. If an older person insists on non-resuscitation, any technologies that alert to a fall or loss of consciousness may be only an increase in caregiving resources that do not serve the autonomous wishes of older persons who have accepted an end to their lives (also see quote 4).

#### Misalignment 2: Wanting more technology-supported care than deemed meaningful by professional carers

Older people appreciated the technologies to call for carers during emergencies and falls or to receive more care, even for those who self-reported to be technology averse. For those who may be more afraid of sudden health-averse incidents occurring when they were alone, technologies could make them feel less anxious. One older person currently living at home described a fall onto a bedside table, where his wife was able to immediately call for emergency services. He lauded the potential benefits for a device for when he were alone, “*because I couldn’t get up on my own, so I really would have had to press a button like that if I had been alone*.” Another older person living at home also addressed the pressure mat for its benefits to catch movements for persons with dementia: *“As soon as they got out of bed, then there was an alarm. I think that’s a clever thing to do.”*

Professional carers nevertheless also questioned the need to monitor activities and health of older persons, particularly those with multiple underlying illnesses or at an advanced age. There is a natural progression of decline and deterioration when one ages, where one’s cognitive and physical abilities would necessarily deviate from the ideal of health or vitality. One professional carer in the nursing home put it: “*…in old age you don't really have to anymore, the question is how much you've already lost, or what you can't do anymore.”* There are risks and accidents that are inevitably present in everyday life, which would be meaningless to prevent if the older persons themselves do not want to be ‘bubble-wrapped’ against (also see box 2, quote 1). Although it would be great to assist them to stay as independent for as long as possible, there would ultimately be the day to let go and let death come ‘as it comes’ (also see box 2, quote 2). Another professional carer in the nursing home posed the rhetorical question: “*Does the senior citizen want this monitoring? What does she get out of it? Does she still want to live at all or is she perhaps […] also want to die?*” In which case, the carer concluded: *“Then maybe I don't necessarily need these devices*.”

Box 2 **Additional quotes**

1. PCH4: I think the basic problem is: “Is it possible and sensible to be able and want to avert every danger? Isn't it part of life that there are certain dangers?”
2. PCN3: I think the question is more, what do I get out of these recordings? What is it really going to help me with? […] It's not like we have some kind of delicate monitoring situation, like in an intensive care unit, where someone starts coughing or vomiting and you have to run immediately and everything, because at the end of the day, they're allowed to live, but they're also allowed to die with us.
3. PCN5: There are also senior citizens who ring the bell, sometimes perhaps for no reason, at least as far as we're concerned. They have some kind of reason and some kind of need and sometimes they can't name it. It can be a fear of some kind, or yes, “I'm lonely at the moment, someone should come” and forget that I rang the bell two minutes ago, but I just want someone there now. And the nursing staff just can't do it and then quickly run into the room to check whether something is there or not. Of course, you can say that it can also be annoying… (laughs) and put a strain on the nursing staff.

For some professional carers, having technology in care does not necessarily make their jobs easier or more reassuring. One professional carer thought it would create additional work and not be suitable for the conditions of the nursing home said: “*I can't imagine in everyday life, when I work with sensors, if something happens to me, an alarm goes off and then I get on my phone* [to check what is wrong].*”* Other professional carers spoke of negative experiences in caregiving, where work would become overwhelming and would require them to distance themselves from certain older persons with higher needs. For older persons with cognitive deterioration, they may become forgetful or confused and summon the carer at a high frequency and for very small tasks. One professional carer expressed that older persons may press the button “*every 5 minutes or 10 minutes, for very small things. ‘Can you scratch my back or can you get the glass for a moment?’ And then after 5 minutes she rings again and asks, can you pour water now? It’s something like that.”* Although the technology was seen as positive *in theory*, it could also be rung at a very frequent interval that could be perceived as ‘annoying’ and result in the nursing staff being on constant alert or strain (see box 2, quote 3).

## Discussion

### Our major findings

Similar to previous literature,^18^,^20^ our study showed ambivalent feedback from end-users towards the use of technologies for alleviating professional care burdens. Nevertheless, deeper analyses reveal that this ambivalence may be connected to the subjective misalignment of older persons’ and their professional carers’ moral outlooks on care, life, death and ageing. In this section, we will address the ethical implications of the misalignment of the expressed attitudes and moral outlooks using the capabilities approach, the sufficiency theory, as well as contemporary discussions of privacy protection, respect for autonomy, and the culturally contingent perceptions of ageing and death.

### Moral outlooks in care and ageing

Diversity exists in our expectations on life, death,^28^ care^29 30^ or ageing that influence the provision or reception of professional care. Professional carers that we interviewed projected their care expectations for their own future selves onto their care recipients, reflecting on what they would themselves appreciate when they were in the places of those that they care for. In a study on a community-based elder care programme, professional carers pointed to the concept of karma that was motivating their work ethic and diligent care provision as this would be how they would like to be treated when they become older.^30^ Older persons also differed in their expectations for professional care depending on the availability of familial or informal care, which may impact the level of assistance and attention they seek from professional carers.^31^ Our qualitative findings further revealed sentiments of pride from older persons for being independent from unnecessary care, fearing being a burden on their carers, as well as considerations from professional carers on enabling older persons to live autonomously. For some, maximising physical safety and health does not necessarily translate to ageing well but is instead dependent on social participation or the making of a meaningful life.^32^ The value on independence and self-sufficiency upheld certain countries, such as those from some of our German-speaking participants, may vastly differ from those with more familial-dependent values of care, which must be taken into account when planning for elder care on a systemic or policy basis.^33 34^ This may be particularly valid in the context of greater global mobility of care resources, such as the flow of foreign care professionals with varying cultural expectations that differ from those held by the German-speaking older care recipients in Switzerland. This subjectivity in preferences and moral outlooks towards care and ageing may reflect the diversity of expectations that one may hold before even implementing the technologies, which would also hint at their later preferences for technology integration.

The implementation of technologies to care for older persons may be seen as opportunities to enhance the intrinsic value of the user, with or without maximising health or the extension of life-years.^35^ In contemporary literature on the status quo of ageing, researchers distinguish between the chronological (life experiences, wisdom, knowledge) and biological (physical deterioration from time lived) types of ageing, which recognises the diversity of expectations that each of us has towards care, ageing, death and decline.^36^,^38^ In other words, the passing of time lived in older age is no longer the sole criterion or dimension of interest of a life well-lived, nor is the pursuit of health and safety for all individuals. In this way, technologies’ role would ideally no longer be solely aimed to fulfil functions of health-promotion, such as ‘reduc(ing users’) to their bodily functions’, but also enhancing the subjective experiences in the ageing process.^39^

### Why is alignment important?

The alignment of preferences of the carer and care receiver could lead to both perceiving the care as good care.^29^ Studies have shown the benefits of a nurturing and satisfactory relationship between the older person and their professional carers, where the needs and values are recognised and met in an individualised manner. Person-centred nursing homes could decrease boredom and levels of depression in older persons and increase satisfaction in the staff.^40^ When there is better understanding between the older person and their professional carers, the carers are rewarded with positive feedback and trust to strengthen their commitment to providing more care to their clients.^30^ On the other hand, a difficult and unsatisfactory caregiving arrangement could lead to burnout for carers, time wasted negotiating with specific clients, neglect to the needs of other clients and themselves, and greater challenges in professional care tasks in general. In our analyses of two misalignments, the professional carers expressed futility with wanting to provide more care to those older persons who did not want to receive more intervention, as well as others that led to frustration and annoyance at being bothered too many times with ‘useless’ alarms. We argue that both represent a misalignment between the expectations of professional care and responsibilities, which was reflected by the use of technologies. Interrogating these findings a step further, there may also be an intrinsic value in finding and respecting an alignment of moral outlooks in receiving and providing professional care. Specifically, the capabilities approach emphasises the importance of allowing individuals freedom to explore and enhance their capabilities to live lives that are deemed uniquely valuable.^41^ Through this lens, any care arrangement that affects the autonomous pursuit of an individual is deemed problematic and inefficient to fully realising one’s capabilities and functioning. In other words, the participants who preferred higher frequencies of professional care and health through technologies should be equally recognised as valid as those preferring to summon care at fewer intervals to enhance their feelings of independence. The autonomous pursuit of recognising and enabling an alignment of moral outlooks could translate to an enhancement of the intrinsic enjoyment of life lived, or care given and received. Similarly, other scholars have applied the approach^42 43^ and noted that health technologies may be a resource that could be used to realise capabilities to provide or receive care (or not). While the benefits to expand capabilities are often advertised clearly by the market, such as the promotion of safety or health are often advertised clearly, their realisation depends on the right conversion factors, which are typically interpreted as personal or environmental characteristics such as preferences, values or conditions.^43^ We argue that the alignment of moral outlooks presented in this paper could also be interpreted as a conversion factor that determines the translation of resources to capabilities and functioning that a person may value. In other words, allowing a professional caregiver to enhance the level of care they value through the technologies is a functioning that depends both on the freedom to exercise this right, as well as the acceptance of older persons. Conversely, allowing an older person to receive care in a way that they value is also fulfilling their own functioning. Therefore, not only is the freedom to pursue capabilities critical in this equation, but also the alignment of moral outlooks in a care arrangement that allows the capabilities of technologies to be fully realised.

### Contributing to the ethics of digital technologies

Balancing the wishes of older persons with the promotion of health often brings in delicate and complex discussions of autonomy and safety, sometimes even privacy concerns as well. Researchers have questioned the ability of digital health technologies that promise to empower users to intrinsically fulfil goals, but rather to discipline users to carry out health-promoting activities to ensure their health.^44^ In some ways, the growing surveillance in health may impinge on our ability to exercise our own freedom, to fully participate in our own lives.^45^ According to digital ethics scholars such as Carissa Véliz, privacy losses may disempower citizens to explore our ability to make decisions intrinsic to our own identities.^45^,^47^

### Setting a limit to alignments

Goldsteen *et al*^28^ argue that while it is important to recognise the diversity of values and moral responses that patients have towards death, professionals should not blindly accept any responses offered by patients. There is a set of normative expectations that enable health professionals to hold normative power towards the patients they care for. Similar to our context of using technologies to alert for professional care support, the matching of subjective preferences should be limited to certain circumstances where it is still within the normative framework of providing ‘good elder care’. That is, there need to be objective limits on what older persons or carers could expect or desire, such as the risks which an older person may be exposed to or what they can demand through technology from the carers. A useful paradigm to guide the implementation of a limit to subjective alignments is the sufficiency theory of justice in health, which includes an objective threshold for the access to adequate health.^48^ This concept of a threshold translates to the normative expectation for the least amount of care offered by professional carers. This mechanism would act as a safeguard against harms for injustices that ensures care recipients access to adequate care, even if both parties subjectively deem it as unnecessary. An actionable policy would be one that may only allow the satisfaction of subjective alignments within a care arrangement only above this threshold, any level of care falling below which binds professional carers to a duty to provide older persons the access to care. For example, if both professional carers and older persons aligned their views to see alerts in emergencies as superfluous and care unnecessary, it may still be pertinent for professional carers to maintain the responsibility of at least guiding the older person to sufficiently explore the meaning of these preferences. The subjective ideal may not always be the objective ideal, and a limit should be set for the professional carer to remain cognizant of the normative expectation of elder care.

### Practical recommendations

Previous studies we published have outlined the possible ways to improve the designs of technologies to enable greater control and protection of user privacy.^8 10 23 26^ As Ratti and Graves^43^ nevertheless write, although the benefits (capabilities) that technologies provide ‘can be easily spelled out,’ the difficulty lies in lining up the conversion factors to enable the technological capabilities to be fully realised in a manner intrinsically valuable to the user. We, therefore, offer recommendations relevant to the implementation of technologies and professional care. Specifically, to have more articulate discussions about the preferences and moral outlooks that individuals involved in care hold intrinsically valuable. The answers to these questions may be related to cultural beliefs, to societal expectations, or to one’s own perception of ageing or dying well. Thereafter, allowing their application into providing care or receiving care in a degree that is autonomous and intrinsic, staying within the bounds of allowing the older persons sufficient access to care. The ability to recognise and express these sentiments true to oneself may require caregiver training programmes, or roundtable discussions with care recipients, in a manner that is practically actionable to each caregiving setting. Nevertheless, it may also be the first critical step to allowing smart home health technologies to more efficiently and holistically support both professional carers and older persons in the ageing process.

### Study limitations

Our paper conducted a secondary analysis of the qualitative data on the attitude of Swiss older persons and professional carers towards smart home health technologies, where we specifically looked at the impacts on professional care. This inevitably influenced the interpretation of our data through these lenses and did not address the specific functions of the technological devices in detail. The older persons and professional carers were not interviewed together, thus were not able to comment directly on a specific professional care arrangement about whether their preferences and values did align. Hence, we do not imply that the older persons or professional carers were referring to their current professional care situations when they expressed dissatisfaction. The qualitative methodology and limited sampling mean a limited generalisability or representativeness of our results. Due to the cost of the technologies, the participants were only shown videos and images of the technologies to elicit their perceptions in their daily use. However, other studies have also gathered attitude and preference information in similar ways.^49 50^

## Conclusions

Under the context of growing caregiving resource shortages and the development of technical solutions for elder care, we presented two ways in which the implementation of assistive and smart monitoring technologies could lead to inefficiencies and dissatisfactions in reducing professional caregiving burdens. In doing so, we broadened the literature on the knowledge and acceptance of technologies by drawing out the link to one’s moral outlooks on care, life, death and the deterioration of age. With these in mind, we argued that an autonomous alignment of these moral outlooks and preference for technologies could be an opportunity to improve professional care burdens and fulfilment of capabilities. Nevertheless, we also caution against the simplification of alignment to these factors and remain aware of an objective expectation of good elder care beyond subjective views. Our findings also contribute to the widening of an understanding of ageing and technologies beyond those filled with passive narratives of decline and care reception for its biological needs, but to recognising and respecting the rich subjective experiences of ageing that may differ from merely promoting the length of life.

## Data Availability

Data are available on reasonable request.

## Footnotes

We have included a small portion of the data exported during this stage in the supplementary files.
